# Needling Interventions for the Management of Musculoskeletal Pain Syndromes

**DOI:** 10.3390/jcm10194603

**Published:** 2021-10-07

**Authors:** César Fernández-de-las-Peñas

**Affiliations:** 1 Department of Physical Therapy, Occupational Therapy, Physical Medicine and Rehabilitation, Universidad Rey Juan Carlos, Alcorcón, 28922 Madrid, Spain; 2 Cátedra Institucional en Docencia, Clínica e Investigación en Fisioterapia: Terapia Manual, Punción Seca y Ejercicio Terapéutico, Universidad Rey Juan Carlos, Alcorcón, 28922 Madrid, Spain

Needling interventions consist of the use of filiform needles for the management of different conditions of the neuromusculoskeletal system. The most commonly used needling therapies are trigger point dry needling, an intervention showing an increasing interest in both clinical and research setting [1], and acupuncture, an intervention used from several centuries ago. In recent years, different dry needling textbooks showing multiple applications of dry needling have been published [2,3]. We present the Special Issue entitled “Needling Interventions for the Management of Musculoskeletal Pain Syndromes” to promote high-quality research on needling interventions. This issue has received special attention, with a total of 12 peer-reviewed papers, nine original articles, one review, and two meta-analyses. These texts describe clinical trials showing the effectiveness of different needling interventions such as dry needling, acupuncture, percutaneous electrolysis (needling combined with galvanic electrical current), and percutaneous electrical nerve stimulation (needling combined with biphasic electrical current) for outcomes ranging from pain to improved motor strength. For instance, García-de-Miguel et al. found that the application of percutaneous electrical nerve stimulation (PENS) on the levator scapulae was more effective than dry needling for improving pressure pain sensitivity and related disabilities in the short term in individuals with neck pain [4]. Two randomized clinical trials demonstrated that the application of percutaneous electrolysis (needling combined with galvanic electrical current) was effective for the treatment of lateral epicondylalgia [5] or supraspinatus tendinopathy [6] when combined with an eccentric exercise program. Gallego-Sendarrubias et al., in a pilot randomized clinical trials, observed that adding two sessions of ultrasound-guided PENS (needling combined with biphasic electrical current) applied on the femoral nerve before a training strength program improves countermovement jump and squat performance speeds in recreational soccer players [7]. On the contrary, the clinical trial by Garrido-Ardila et al. revealed that acupuncture did not exert significant improvements in health-related quality of life, pain, joint stiffness, difficulty to work, and depression in women with fibromyalgia syndrome [8]. Finally, in a preliminary trial, Pérez-Bellmunt et al. found that dry needling was able to change muscle tone, relaxation, pressure pain sensitivity, and creep when applied over trigger points in the lateral gastrocnemius [9]. In addition to these clinical trials, a milestone paper using an animal model revealed the molecular effect of a particular needling intervention, e.g., percutaneous electrolysis [10]

Two studies focused on the use of ultrasound imaging: the first one for improving the safety of some potential dangerous dry needling treatments when applied on the thorax [11] and the second one suggesting that dry needling influences muscular morphology by decreasing masticatory muscle thickness as assessed with ultrasound imaging [12]

In addition, two meta-analyses support the effectiveness of trigger point dry needling for conditions that cause neck [13] and knee pain [14]. Finally, a scoping review revealed that neuropathic pain mechanisms are not routinely considered in needling approaches when treating individuals with sciatica [15]. Current and previous evidence support the effectiveness of needling interventions for the management of chronic pain conditions and the need to integrate these techniques into updated neuroscience paradigms [16].

## Data Availability

Not applicable.
